# Reliable transfer of transcriptional gene regulatory networks between taxonomically related organisms

**DOI:** 10.1186/1752-0509-3-8

**Published:** 2009-01-15

**Authors:** Jan Baumbach, Sven Rahmann, Andreas Tauch

**Affiliations:** 1 International Computer Science Institute, Berkeley, CA 94704, USA; 2 Bioinformatics for High-Throughput Technologies, Technical University of Dortmund, D-44227 Dortmund, Germany; 3 Institute for Genome Research and Systems Biology, Center for Biotechnology, Bielefeld University, D-33594 Bielefeld, Germany

## Abstract

**Background:**

Transcriptional regulation of gene activity is essential for any living organism. Transcription factors therefore recognize specific binding sites within the DNA to regulate the expression of particular target genes. The genome-scale reconstruction of the emerging regulatory networks is important for biotechnology and human medicine but cost-intensive, time-consuming, and impossible to perform for any species separately. By using bioinformatics methods one can partially transfer networks from well-studied model organisms to closely related species. However, the prediction quality is limited by the low level of evolutionary conservation of the transcription factor binding sites, even within organisms of the same genus.

**Results:**

Here we present an integrated bioinformatics workflow that assures the reliability of transferred gene regulatory networks. Our approach combines three methods that can be applied on a large-scale: re-assessment of annotated binding sites, subsequent binding site prediction, and homology detection. A gene regulatory interaction is considered to be conserved if (1) the transcription factor, (2) the adjusted binding site, and (3) the target gene are conserved. The power of the approach is demonstrated by transferring gene regulations from the model organism *Corynebacterium glutamicum *to the human pathogens *C. diphtheriae*, *C. jeikeium*, and the biotechnologically relevant *C. efficiens*. For these three organisms we identified reliable transcriptional regulations for ~40% of the common transcription factors, compared to ~5% for which knowledge was available before.

**Conclusion:**

Our results suggest that trustworthy genome-scale transfer of gene regulatory networks between organisms is feasible in general but still limited by the level of evolutionary conservation.

## Background

In the post genome era we observe a continuously growing, vast amount of sequenced organisms spread over all domains of life. Besides the identification and annotation of functional sites within the emerging nucleic acid sequences, an important task in molecular genetics, biotechnology, and human medicine is to unravel the regulation of these sites. DNA-binding transcription factors (TFs) are the most important components of the cell's regulatory machinery [1]. They recognize specific operator sequences close-by the promoter regions of the controlled target genes, referred to as transcription factor binding sites (TFBSs), and thereby influence the amount of produced proteins. Although inevitable for the understanding of the cell's handling of changing environmental conditions, the wet-lab reconstruction of the resulting transcriptional regulatory networks is cost-intensive, time-consuming, and impossible to perform for any species separately [2,3]. Even for prokaryotic model organisms, such as *Escherichia coli *or *Corynebacterium glutamicum *the monumental task of deciphering transcriptional regulatory networks for whole species is far from being complete. The current knowledge is brought together and stored in reference databases, such as RegulonDB [4] and CoryneRegNet [5]; see [6] for a more detailed analysis of such platforms.

The gathered information about substantial parts of the transcriptional regulatory apparatus is used to study conserved network structures, sensing mechanisms, and to uncover hidden architectures behind gene regulatory networks [7,8]. In addition, specialized approaches, based on the evolutionary conservation of the responsible transcription factors and the controlled target genes, are used to transfer knowledge on gene regulatory networks between different organisms but aim to provide more general, qualitative conclusions (trends) across many species [9]. The main problem, however, is the neglect of the fact that orthologous regulators and target genes not necessarily are involved in conserved regulations. Another complicacy is the dependency on reliable homology detections. Other approaches utilize annotated transcription factor binding sites to compute mathematical models for further TFBS predictions; where the by far most widely used model for TFBSs are position weight matrices (PWMs) [10]. Here, the major intricacy lies in the comparatively low level of TFBS conservation between different organisms [11], even for essential factors such as the bacterial SOS response and DNA damage regulator LexA [12]. Hence, the consideration of PWM-based predictions apart from further evidence is not very meaningful. Moreover, there is a hidden problem with PWM calculations: The determination of the position to which a transcription factor binds is difficult and time-consuming. It is normally performed through electrophoretic mobility shift assays, DNAse footprinting, ChIP-to-chip assays, or mutations of putative TFBSs [13-15]. With all of these methods a precise identification that is accurate to one base pair is problematic. Furthermore, since TFs bind the double-stranded DNA it is a matter of interpretation which strand of the DNA sequence is annotated and stored in the database. This causes a practical problem when a motif from either strand based on approximate knowledge of its position is used for PWM construction.

## Methods

In the past years, we extensively studied the transcriptional regulatory repertoire of the model organism *C. glutamicum *and other corynebacteria important in human medicine and biotechnology. We gathered all publicly available data, combined it with own wet-lab findings and developed the reference database and analysis platform CoryneRegNet [5,11,12,16]. Here we introduce an integrative bioinformatics approach that aims for a reliable transfer of gene regulatory interactions, which combines both of the above introduced major approaches: homology detection and DNA binding site prediction. Instead of studying general trends and conserved network motifs across hundreds of organisms we are interested in high-quality predictions with just few or even no false-positives for *C. diphtheriae*, *C. efficiens*, and *C. jeikeium *based on evidenced observations from the model organism *C. glutamicum*.

**Figure 1:**
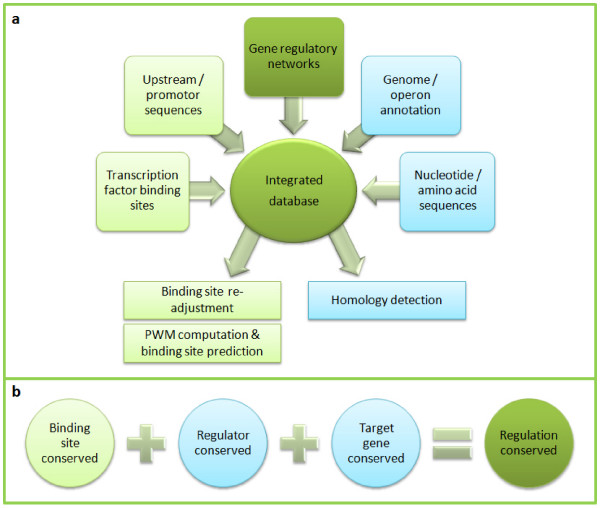
**Scheme of the transfer workflow**. **a**, Simplified structure of a typical transcriptional gene regulatory interaction database. Using genetic upstream sequences and transcription factor binding site annotations the TFBSs can be re-adjusted and modeled as PWMs for subsequent TFBS predictions. Sequence clustering tools can be applied to the stored genome annotation and gene/protein sequences to gain information about homologous genes/proteins. **b**, A regulatory interaction can be transferred from a model organism to a closely related species if the regulator as well as the target gene are orthologous and a matching TFBS can be found in the upstream sequence of the orthologous target gene.

The success of the approach relies on the optimal interplay of the used bioinformatics components. On a very general level, the workflow is depicted in figure 1. An integrated database system is used for data fusion of annotated nucleotide and amino acid sequences together with evidenced transcriptional regulatory relationships and corresponding sequence features. By having all required data at hand we can re-adjust inaccurately determined TF binding sequences by shifting some motifs by some positions and by assigning a strand annotation, if necessary. Sequence motif discovery tools [17] may be utilized for that purpose or publicly available special purpose tools, such as MoRAine [18]. After computing a PWM for each transcriptional regulator of the model organism from the re-annotated TFBSs, motif matching tools (such as PoSSuMsearch [19] or MATCH [20]) are used to predict binding sites in the target organism assuming that the DNA-binding motif of the regulator is sufficiently conserved. This is done for any pair of the conserved regulators and target genes. It is obvious that the TF itself has to be conserved in the target organism; but the second condition (homologous target genes) is very important as well to provide reliable, high-quality predictions since a high-scoring PWM-based sequence match alone is not very meaningful [10]. This is the only way to reduce the huge amount of false-positives without further background knowledge even for comparatively restrictive thresholds that miss most of the true-positives. An application example illustrating the problem for corynebacteria may be found in ref. [11]. The detection of conserved, orthologous proteins based on the given amino acid sequences alone was a long-standing challenge in computational biology. It emerged that clustering approaches utilizing BLAST[21]-based pairwise sequence similarity measures attack the problem comparatively well. Popular approaches are Markov clustering [22], spectral clustering [23] and graph cluster editing [24,25].

## Results and discussion

**Figure 2:**
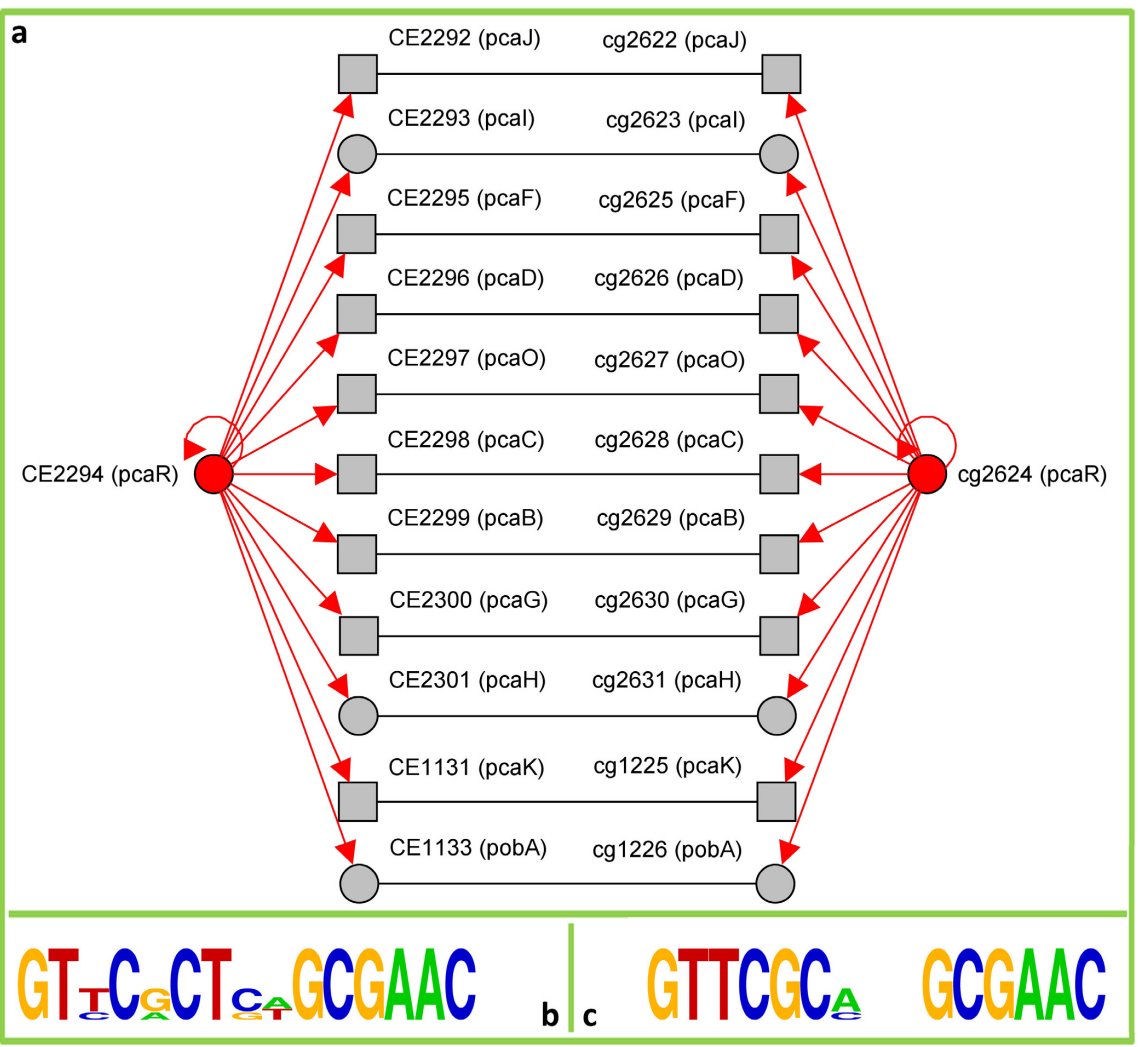
**Illustration of the gene regulatory network for PcaR**. **a**, A comparative visualization of the known gene regulatory network of PcaR in the model organism *C. glutamicum *(right) transferred to *C. efficiens *(left). Nodes correspond to genes, directed (red) edges to negative transcriptional regulatory interactions, and undirected (black) edges to a sequence-based similarity that indicates a putative homology. **b/c**, The sequence logos computed from the PcaR binding sites in *C. efficiens *(predicted, left) and in *C. glutamicum *(evidenced, right).

To demonstrate our combined approach we decided to integrate MoRAine [18] for TFBS readjustment, PoSSuMsearch [19] for binding motif matching, and FORCE [25] for homology detection and applied it to corynebacterial data gained from CoryneRegNet [5] (see additional file 1). The goal was to use proven knowledge from the model organism *C. glutamicum *(*CG*) to predict reliable gene regulatory networks in *C. diphtheriae *(*CD*), which is a human pathogen, *C. jeikeium *(*CJ*) that is pathogen and extremely antibiotic resistant, and the sister organism of *CG* namely *C. efficiens *(*CE*), which is import for biotechnological production processes. First, the homology detection identified the common repertoire of conserved transcription factors between *CG *and *CD*, *CE*, and *CJ *(49, 77, and 31 regulators respectively). This observation of an average common TF set of ~70% fits well with the previously published study on the individual and common repertoire of corynebacteria [26]. For the next step in the pipeline the known transcription factor binding sites for each of the 69 characterized TFs of *CG *have been re-adjusted resulting in an average improvement of the mean information content of the underlying position frequency matrices of ~23% (see additional file 2). Subsequently, PWMs are computed and matched with all upstream sequences of orthologous target genes in *CD*, *CE*, and *CJ *to predict putative TFBSs. In the case of ambiguity of the homology prediction, the pipeline also considers the neighborhood of the respective genes in the target organism and chooses the candidate gene with the maximal number of homologous genes in the genetic surrounding. If the regulator, the target gene, and the binding site are conserved sufficiently, the regulation is considered to be conserved and added to the pipeline's output. Although these restrictions are very strict, we found 530 reliable novel gene regulations for *CD*, *CE*, and *CJ *and thereby increased the database content of the reference database CoryneRegNet considerably (factor 4.2). For the three organisms we identified reliable transcriptional gene regulations for ~40% of the common transcription factors, compared to ~5% for which knowledge was available before. Table 1 statistically summarizes the original and the transferred database content. Figure 2a exemplarily shows a network visualization of the PcaR regulon as known from *CG *compared to the transferred one of *CE*. It is obvious that all of the 11 target genes including the regulator itself are orthologous between *CG* and *CE*. Since the binding sites are conserved too, as indicated by the sequence logos in figure 2b/c, the pipeline considered all regulations as transferable from *CG *to *CE*. This observation fits well with the known PcaR regulon of *C. efficiens *[27].

**Table 1:** Comparison of the original and the transferred database content of CoryneRegNet.

	TFs	TFs_C_	TFs^K^		TFs_C_^K^	Regulations	
CG	128		69			530	

			original	transferred	transferred	original	transferred

CD	63	49 (77.8%)	2 (3.2%)	20 (×10)	20 (40.1%)	46	193 (×4.2)

CE	103	77 (74.8%)	5 (4.9%)	28 (×5.6)	28 (36.4%)	64	348 (×5.4)

CJ	55	31 (56.4%)	1 (1.8%)	13 (×13)	13 (41.9%)	51	150 (×2.9)

Av		69.6%	3.3%	x9.5	39.7%		x4.2

Abrev.: CG = The model organism *Corynebacterium glutamicum*, CD = *C. diphtheriae*, CE = *C. efficiens*, CJ = *C. jeikeium*, Av = Average, TFs = Transcription factors, TFs_C _= Common transcription factors with *C. glutamicum*, TFs^K ^= Transcription factors with knowledge, TFs_C_^K ^= Transcription factors common with *C. glutamicum *with knowledge, Regulations = Transcriptional regulatory interactions, original = Original database content, transferred = Database content after network transfer. Percentages/factors: TFs_C _= relative to column TFs, TFs^K^/original = relative to column TFs, TFs^K^/transferred = relative to column TFs^K^/orig., TFs_C_^K ^= relative to column TFs_C_, Regulations/transferred = relative to column Regulations/original.

The whole data analysis procedure is very time-efficient (< 1 min computing time) and we added the new datasets into the CoryneRegNet 5.0 database for public access. Although it is very likely that our predictions are highly reliable we separated the evidenced from the predicted datasets within the front-end and now provide two sub-versions of CoryneRegNet.

In the application described above, we combined the software packages MoRAine, FORCE, and PoSSuMsearch into a data fusion workflow that is responsible for the data transfer between model and target organisms. One could also think about a combination of other computational biology tools designed for the same purpose. For instance FORCE could be replaced by TribeMCL [22] and PoSSuMsearch by MATCH [20]. We decided to use PoSSuMsearch since it provides statistically sound p-value computations within reasonable response times [19]. FORCE has been included into the pipeline since it has been shown to outperform other clustering approaches on prokaryotic datasets [25].

With Regulogger [28], Alkema *et al*. presented a pipeline that mainly focuses on the reconstruction of conserved regulatory networks for *Staphylococcus aureus*. Regulogger mainly concentrates on the detection and characterization of conserved sequences in promoter regions (phylogenetic footprinting) of orthologous genes. For the determination of these orthologous genes, the COG database [29] is used, which is impracticable at least for our target species since no COG annotations are available for other corynebacteria than *C. glutamicum*. In the near future, novel ultra-fast sequencing technologies will provide much more data on organisms that are not included in COG. In [30] and a subsequent follow-up study [31], the TRACTOR_DB database [32] was used to identify conserved regulatory interactions between *E. coli *and thirty gamma-proteobacteria. Here, pairwise BLAST Bidirectional Best Hits (BBHs) are used directly to identify orthologous genes, which may result in suboptimal predictions [25]. However, the presented results support our strategy to combine orthology information with binding site detection. Besides, the conservation of gene regulatory networks between corynebacterial organisms has never been studied before and the integration of the presented results into the CoryneRegNet reference database provides a powerful tool for further network studies.

The current study is strongly limited by the level of phylogenetic conservation between the reference organism and the target species. Since remodeling of transcriptional gene regulation is a crucial strategy used by bacteria to evolve and regulate novel biochemical features, (1) specific regulatory pathways may have been altered and (2) unique transcriptional regulatory mechanisms may have been developed. The following three limitations with our approach for inter-species network transfer arouse: (1) Interactions that do not evolve in the reference species cannot be detected in a specific target organism. (2) Utilizing pure sequence-based similarities for *in silico *orthology detections neglect that proteins with comparably high overall amino acid sequence similarity may have different specific functions within the cell although they are predicted as putative homologs and vice versa. (3) In our approach, we assume that a conserved transcription factor in the target organism interacts with a binding site that is very similar to that in the reference organism. This is not necessarily the case and may result in both, false positive and false negative predictions. However, the rapidly increasing amount of fully sequenced organisms without much background knowledge about their gene regulatory repertoire strongly restricts our alternatives to computational methods that utilize sequence-based evolutionary conservation.

## Conclusion

Besides the immediate advantages for the worldwide medical and biotechnological corynebacteria community, we anticipate the presented results to be a starting point for an integrated analysis of gene regulatory networks in the light of a combined analysis of orthologous genes conjointly with conserved DNA binding sites. Although we tested the presented strategy with corynebacteria, it is of general interest and can be applied to many other organisms as well. We conclude that trustworthy transfers of gene regulatory networks between organisms on a genome-scale are feasible but still limited by the level of evolutionary conservation.

## Availability and requirements

Project name: CoryneRegNet 5.0

Project home page: 

Operating system(s): Platform independent

Programming language: PHP, Java 6

License: Academic Free

License (AFL)

Any restrictions to use by non-academics: No.

## Authors' contributions

JB designed and implemented the data analysis and transfer pipeline. SR and AT co-supervised the project, host the web services, and curate the database content.

## Appendix

Here, we briefly introduce the integrated bioinformatics tools that have been utilized for this article.

### MoRAine – Regulatory binding site re-adjustment

MoRAine is a software that re-assesses and re-annotates transcription factor binding sites. Each TFBS sequence with experimental evidence underlying a PWM model is compared against each other. MoRAine heuristically solves a combinatorial optimization problem to readjust TFBSs by possibly switching their strands and shifting them a few positions to the left or to the right in order to maximize the mean information content of the resulting PWM. In [18] we validated and confirmed the improvement of the PWM-based TFBS prediction performance for *E. coli *by using MoRAine as pre-processing step prior to PWM computation. For this article, we applied MoRAine to adjust corynebacterial TFBSs for computing the PWM model being the input for PoSSuMsearch.

### PoSSuMsearch – Statistically sound binding site prediction

The *in silico *prediction of TFBSs is a long-standing challenge in computational biology and several software tools exist for this purpose. Here we used PoSSuMsearch. It provides a combination of (non-permuted) lookahead scoring and efficiently searching an enhanced suffix array that previously has been created from corynebacterial upstream sequences. The scores of putative matches are compared to a threshold that is computed based on the tolerable frequency of hits in random sequences (p-value) by an efficient and exact lazy-evaluation method [19]. To our knowledge, PoSSuMsearch is the only available bioinformatics tool that provides a statistically sound TFBS match evaluation together with reasonable response times. We used PoSSuMsearch to predict putative binding sites for conserved corynebacterial regulators.

### FORCE – Transitivity clustering of protein sequences

Another long-standing challenge in bioinformatics is the detecting of homologous proteins based on amino acid sequence similarity. For this purpose, we recently developed a clustering approach based on weighted graph cluster editing (or weighted transitive graph projection). With FORCE, we presented a heuristic that solves the respective NP-hard graph-modification problem. In [25], we demonstrated the ability of FORCE to cluster hundreds of thousands of protein sequences efficiently and accurately. Our evaluations with gold standard databases show that it outperforms other tools at least in terms of accuracy. In particular its ability to handle huge datasets makes it the ideal candidate for the study introduced in this article. We used FORCE as homology detection software to identify conserved transcriptional regulators as well as orthologous target genes.

## Supplementary Material

Additional File 1 **Data fusion workflow as used for the presented study with CoryneRegNet.** We used all validated datasets of *Corynebacterium glutamicum *from the CoryneRegNet database. All known transcription factor binding sites were re-adjusted by using MoRAine (no position shifts, but strand annotation; method: Cluster growing/Motif-seed similarity) for subsequent computations of position weight matrices (PWMs). We utilized PoSSuMsearch to scan the upstream/promoter sequences of all transcription units of *C. efficiens*, *C. diphtheriae*, and *C. jeikeium*, extracted from CoryneRegNet, to scan for putative transcription factor binding sites by using the MoRAine-adjusted binding sites of *C. glutamicum*. All amino acid sequences of *C. efficiens*, *C. diphtheriae*, *C. glutamicum*, and *C. jeikeium *were extracted from CoryneRegNet and grouped into clusters of orthologous/conserved corynebacterial proteins by using the FORCE software. We consider two genes as orthologous/conserved (1) if the corresponding proteins are in the same FORCE cluster and (2) if at least one of the surrounding genes is also "FORCE-conserved". We consider a gene regulatory interaction as conserved between *C. glutamicum *and another corynebacterium if (1) the transcription factor is conserved (2) its binding sites are conserved, and (3) the putative target genes are conserved as well. The corresponding gene regulation is added to the CoryneRegNet 5.0 p database. Refer to the CoryneRegNet web site for an interactive version of this picture including links to the corresponding tools. Click here for file

Additional File 2 **Original and re-adjusted corynebacterial transcription factor binding sites**. The RAR-file contains a list of files, two for each regulator of *C. glutamicum*. They contain the original and the MoRAine-adjusted transcription factor binding sites in FASTA-format. Click here for file
