# Supplementary figures and images for: Reliable transfer of transcriptional gene regulatory networks between taxonomically related organisms
Source: BMC Syst Biol. 2009 Jan 15;3:8. doi: 10.1186/1752-0509-3-8 (PMC2653031; doi:10.1186/1752-0509-3-8)

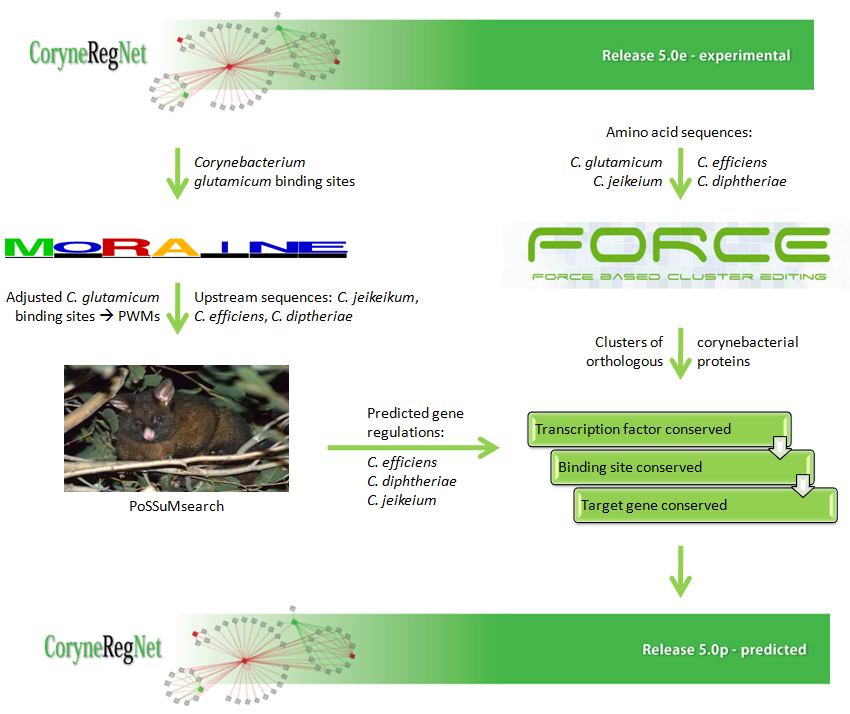

Supplement: Additional File 1 — Data fusion workflow as used for the presented study with CoryneRegNet. We used all validated datasets of Corynebacterium glutamicum from the CoryneRegNet database. All known transcription factor binding sites were re-adjusted by using MoRAine (no position shifts, but strand annotation; method: Cluster growing/Motif-seed similarity) for subsequent computations of position weight matrices (PWMs). We utilized PoSSuMsearch to scan the upstream/promoter sequences of all transcription units of C. efficiens, C. diphtheriae, and C. jeikeium, extracted from CoryneRegNet, to scan for putative transcription factor binding sites by using the MoRAine-adjusted binding sites of C. glutamicum. All amino acid sequences of C. efficiens, C. diphtheriae, C. glutamicum, and C. jeikeium were extracted from CoryneRegNet and grouped into clusters of orthologous/conserved corynebacterial proteins by using the FORCE software. We consider two genes as orthologous/conserved (1) if the corresponding proteins are in the same FORCE cluster and (2) if at least one of the surrounding genes is also "FORCE-conserved". We consider a gene regulatory interaction as conserved between C. glutamicum and another corynebacterium if (1) the transcription factor is conserved (2) its binding sites are conserved, and (3) the putative target genes are conserved as well. The corresponding gene regulation is added to the CoryneRegNet 5.0 p database. Refer to the CoryneRegNet web site for an interactive version of this picture including links to the corresponding tools. [file 1752-0509-3-8-S1.jpeg]
